# Biological Evaluation of Naproxen–Dehydrodipeptide Conjugates with Self-Hydrogelation Capacity as Dual LOX/COX Inhibitors

**DOI:** 10.3390/pharmaceutics12020122

**Published:** 2020-02-03

**Authors:** Rute Moreira, Peter J. Jervis, André Carvalho, Paula M. T. Ferreira, José A. Martins, Patrícia Valentão, Paula B. Andrade, David M. Pereira

**Affiliations:** 1 REQUIMTE/LAQV, Laboratório de Farmacognosia, Departamento de Química, Faculdade de Farmácia, Universidade do Porto, R. Jorge Viterbo Ferreira, n 228, 4050-313 Porto, Portugal; rutemartinsmoreira@hotmail.com (R.M.); valentao@ff.up.pt (P.V.); pandrade@ff.up.pt (P.B.A.); 2 Centre of Chemistry, University of Minho, Campus de Gualtar, 4710-057 Braga, Portugal; andrefcarvalho95@gmail.com (A.C.); pmf@quimica.uminho.pt (P.M.T.F.); jmartins@quimica.uminho.pt (J.A.M.)

**Keywords:** anti-inflammatory, hydrogel, dehydrodipeptide, cyclooxygenase, lipoxygenase, cancer, proteasome

## Abstract

The use of peptide–drug conjugates is emerging as a powerful strategy for targeted drug delivery. Previously, we have found that peptides conjugated to a non-steroidal anti-inflammatory drug (NSAID), more specifically naproxen–dehydrodipeptide conjugates, readily form nanostructured fibrilar supramolecular hydrogels. These hydrogels were revealed as efficacious nano-carriers for drug delivery applications. Moreover, the incorporation of superparamagnetic iron oxide nanoparticles (SPIONs) rendered the hydrogels responsive to external magnetic fields, undergoing gel-to-solution phase transition upon remote magnetic excitation. Thus, magnetic dehydrodipeptide-based hydrogels may find interesting applications as responsive Magnetic Resonance Imaging (MRI) contrast agents and for magnetic hyperthermia-triggered drug-release applications. Supramolecular hydrogels where the hydrogelator molecule is endowed with intrinsic pharmacological properties can potentially fulfill a dual function in drug delivery systems as (passive) nanocariers for incorporated drugs and as active drugs themselves. In this present study, we investigated the pharmacological activities of a panel of naproxen–dehydrodipeptide conjugates, previously studied for their hydrogelation ability and as nanocarriers for drug-delivery applications. A focused library of dehydrodipeptides, containing *N*-terminal canonical amino acids (Phe, Tyr, Trp, Ala, Asp, Lys, Met) *N*-capped with naproxen and linked to a *C*-terminal dehydroaminoacid (ΔPhe, ΔAbu), were evaluated for their anti-inflammatory and anti-cancer activities, as well as for their cytotoxicity to non-cancer cells, using a variety of enzymatic and cellular assays. All compounds except one were able to significantly inhibit lipoxygenase (LOX) enzyme at a similar level to naproxen. One of the compounds **4** was able to inhibit the cyclooxygenase-2 (COX-2) to a greater extent than naproxen, without inhibiting cyclooxygenase-1 (COX-1), and therefore is a potential lead in the search for selective COX-2 inhibitors. This hydrogelator is a potential candidate for dual COX/LOX inhibition as an optimised strategy for treating inflammatory conditions.

## 1. Introduction

Inflammatory diseases affect millions of people all over the world, having severe consequences on their quality of life. Nowadays, there are two types of anti-inflammatory drugs used in therapeutics: steroidal anti-inflammatory drugs and non-steroidal anti-inflammatory drugs (NSAIDs). The challenge of drug delivery is to transport enough drug molecules to the target sites, whilst minimizing adverse effects in healthy and non-target tissues. Two main approaches in this field have been exploited: (1) the use of a delivery vehicle, such as nanoparticles [1,2,3,4,5,6], and (2) the covalent modification of a drug with a small moiety, such as peptide–drug conjugates [7,8,9]. Peptide–drug conjugates are a conventional class of therapeutic agent that are formed through covalent attachment of specific peptide sequences to established drugs via suitable linker and linkage strategies. The conjugation of peptide epitopes of cell receptors to drug molecules allows the targeted delivery of drugs to specific cells and tissues. The vehicle peptide ideally should not detrimentally affect the pharmacological properties of the conjugate. Alternatively, when the conjugate does not retain the pharmacological properties of the drug, it is necessary to ensure that the drug molecule is released at the therapeutic site via a specific stimulus, generally the enzymatic cleavage of the connecting linker. This release step adds an extra level of specificity and safety to the targeted drug-delivery system. In other cases, the peptide endows the conjugate with self-assembly properties that result in improved drug efficacy, e.g., enhanced enzymatic stability of the aggregates or drug nanostructures [7]. 

The local administration of drugs that can act directly at the disease site presents many advantages over systemic delivery, such as increased bioavailability, reduced off-target and adverse effects, and lower cost. The COX enzymes initiate the arachidonic acid metabolic cascade, leading to the formation of pro-inflammatory prostaglandins and thromboxanes. COX inhibitors are good candidates for treating acute and chronic pain through topical application, since it is well known that their prolonged systemic use can produce several side effects, e.g., gastrointestinal, blood clotting and kidney issues, arising from COX-1 inhibition, and cardiovascular problems, arising from COX-2 inhibition [10,11]. Thus, there is a need to modulate the selectivity of these drugs according to their target and to minimize the use of anti-inflammatory drugs through systemic delivery [12]. 

Hydrogels have emerged in recent years as promising carriers for drug delivery applications owing to their easy preparation, compliance by the patient, and their biocompatibility and biodegradability [13]. Hydrogels made of biodegradable polymers have frequently served as carriers to encapsulate therapeutic agents, allowing their controlled release [14]. Drug release can be tuned by adjustment of the pore sizes, incorporation of micro- and nanoparticles, or through the cleavage of covalent or non-covalent bonds [15]. Drug molecules can also be incorporated into supramolecular nanostructured hydrogels through non-covalent interactions. The reversible nature of the weak noncovalent interactions ensures sustained drug delivery and hydrogel’s responsiveness to environmental stimuli [16]. 

The main limitation of supramolecular hydrogels is the proteolytic sensitivity of the peptide hydrogelator molecules by endogenous proteases [17]. One of the strategies reported to overcome this limitation is to replace canonical amino acids with non-proteinogenic analogues, such as *D*-amino acids, β-amino acids or dehydroamino acids [18]. Conjugates of naproxen with peptides containing *D*-amino acids are efficacious hydrogelators with resistance to proteolysis. Importantly, the *D*-amino acid conjugates display enhanced selectivity for the COX-2 isozyme in relation to the unconjugated drug naproxen, a non-selective inhibitor of both COX isozymes [12]. 

Dehydroamino acid residues are commonly encountered in drug discovery, with plinabulin, thiostrepton, imipenem and cilastatin being medicinally important molecules [19,20,21]. In our laboratory, we have investigated peptides containing dehydroamino acids (dehydrophenylalanine- ΔPhe, dehydroalanine-ΔAla, dehydroaminobutyric acid-ΔAbu) as alternatives to *D*-amino acids, not only to provide proteolytic stability but also for the reduced conformational flexibility of the peptide backbone. We have recently reported that dehydrodipeptides capped on the *N*-terminus with naproxen self-assemble into nanostructured hydrogels [18,22]. These peptide conjugates were found to be resistant to proteolysis by chymotrypsin, whereas the corresponding canonical dipeptide–naproxen conjugates undergo proteolysis readily under the same conditions. We have also shown that naproxen *N*-capped dehydrodipeptides, further conjugated with known peptide bioepitopes, such as GRDGD, can also produce hydrogels [17]. Furthermore, we have been able to incorporate drug molecules into dehydropeptide-based hydrogels and demonstrated their sustained drug-delivery properties, thus identifying this type of supramolecular hydrogel as potential nano-carriers in drug delivery systems [17]. Superparamagnetic iron oxide nanoparticles (SPIONs) can also be incorporated into the dehydrodipeptide-based hydrogel networks, providing concentration-dependent T2-MRI contrast enhancement. Upon magnetic excitation, the SPIONs generate heat, which causes the hydrogel to undergo a gel-to-solution phase transition. This means that magnetic hyperthermia can potentially be used as a remote trigger for the temporally and spatially controlled release of contrast agents [23].

**Figure 1:**
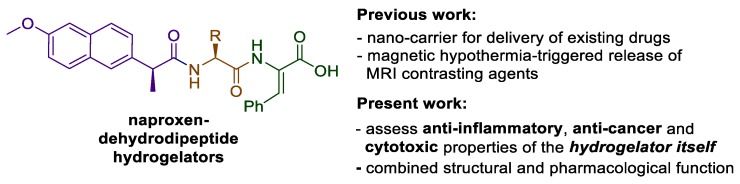
General structure of the hydrogelators to be studied.

**Figure 2:**
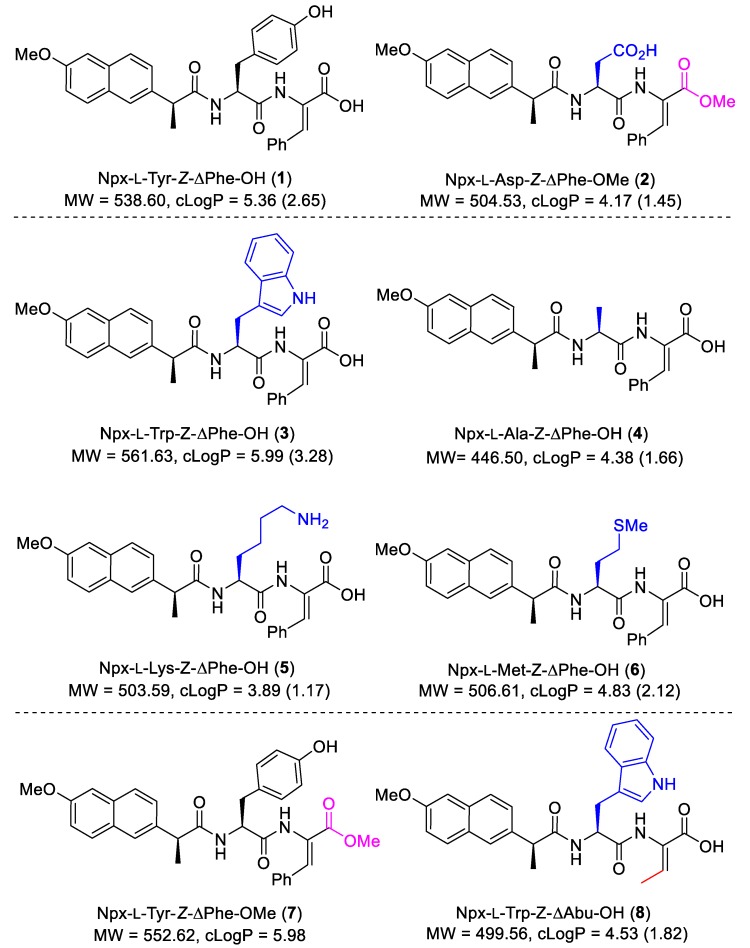
Library of compounds **1**–**8,** evaluated for anti-inflammatory and anti-cancer activity. The molecular weight and cLogP data are shown (values in parenthesis refer to the carboxylate salt form).

While our previous work has focused on the ability of the hydrogels to act as delivery agents for incorporated drugs or diagnostic agents, we have also been keen to investigate the potential of naproxen–dehydrodipeptide conjugates as therapeutic agents by themselves, with obvious added potential for highly targeted topical applications as hydrogels (Figure 1). Usually, drug–peptide conjugates require enzymatic proteolysis to release the active drug from the peptide vehicle. Interestingly, the proteolytic stability imparted by the dehydroamino acid residue presumably allows the naproxen–dehydrodipeptide conjugates to combine the anti-inflammatory properties of naproxen with the administrative benefits of a gel, thus merging structural function with intrinsic pharmacological activity. Alternatively, the conjugates could display novel pharmacological properties not associated with the naproxen moiety. In a preliminary study [23], compounds **1** and **2** (Figure 2) showed a modest ability to inhibit lipopolysaccharide (LPS)-induced production of nitric oxide (^•^NO) radicals (a biomarker for inflammation) in RAW 264.7 cells. These compounds were also tested in enzymatic assays for their ability to inhibit key enzymes in the inflammatory cascade. Compounds **1** and **2** displayed modest levels of COX inhibition (with a low selectivity towards COX-2 inhibition) at 25 µM and high levels of lipoxygenase (LOX) inhibition at 100 µM. 

These initial results prompted us to further explore the structure–activity relationship (SAR) around the naproxen–dehydrodipeptide molecular scaffold (Figure 1) and conduct a detailed investigation of the anti-inflammatory, anti-cancer and cytotoxicity properties of a focused library of naproxen–dehydrodipeptides (Figure 2). 

## 2. Materials and Methods 

### 2.1. Standards and Reagents

Dimethyl sulfoxide (DMSO), ethanol and 2-propanol were obtained from Fischer Scientific (Loughborough, UK). Naproxen, quercetin, sulphanilamide, N-(1-naphthyl)ethylenediamine, 3-(4,5-dimethylthiazol-2-yl)-2,5-diphenyltetrazolium bromide (MTT), LPS of *Salmonella enterica*, linoleic acid, LOX from glycine max (soybean) and trypan blue were obtained from Sigma-Aldrich (St. Louis, MO, USA). COX fluorescent inhibitor screening assay kit was purchased from Cayman chemicals (Ann Arbor, MI, USA). Dulbecco’s Modified Eagle Medium (DMEM), Minimum Essential Medium (MEM), heat inactivated foetal bovine serum (FBS), Pen-Strep solution (penicillin 5000 units mL^−1^ and streptomycin 5000 mg mL^−1^) and trypsin were obtained from Gibco Invitrogen TM (Grand Island, NY, USA). Proteasome 20S, lactacystin and Suc-Leu-Leu-Val-Tyr-AMC were purchased from Enzo Life Sciences. Proteasome 26S was obtained from AGS cells, as described below.

### 2.2. Compounds Tested

The chemical synthesis and characterisation data for the compounds **1**–**4**, **7** and **8** have been described previously [17,18,23]. The synthesis and characterisation data for compounds **5** and **6** are described in articles submitted for publication. The partition coefficient between water and n-octanol (Log P) of each compound was estimated using Molinspiration Cheminformatics software (Molinspiration, Slovensky Grob, Slovak Republic, 2017, http://www.molinspiration.com), as a sum of fragment-based contributions and correction factors, and it is used as quantitative descriptor of compound lipophilicity [24].

### 2.3. Lipoxygenase Glycine Max (Soybean) Assay

The inhibitory effect on LOX was assessed in 96-well plates, using a modified version of a previously reported procedure from Pereira et al. [25]. The compounds were tested in a reaction mixture of each compound (20 µL), phosphate buffer (200 µL, pH 9.0) and soybean LOX (20 µL, ~100 U). After 5 min pre-incubation at room temperature, the reaction was started by addition of linoleic acid substrate (20 µL of a 4.18 mM solution in ethanol). The reaction was monitored at 234 nm using a multiplate reader (Multiskan Thermo Fisher Scientific Oy, Vantaa, Finland), for 3 min.

### 2.4. Cyclooxygenase-1 (COX-1) and Cyclooxygenase-2 (COX-2) Inhibition Assay

The assay was performed using the COX fluorescent inhibitor screening assay kit (Cayman chemical, MI, USA), with some modifications. Briefly, 60 µL of assay buffer (100 mM Tris-HCl, pH 8.0), 5 µL of hemin, 5 µL of enzyme (either COX-1 or COX-2) and 5 µL of compound (25 µM) were added to a black 96-well plate. After 5 min of incubation at room temperature, 5 µL of 10-acetyl-3,7-dihydroxyphenoxazine (ADHP) and 20 µL of a solution containing arachidonic acid (0.5 mM) and KOH (2.5 mM) were added to each well. After a further 2 min at room temperature, the fluorescence of resorufin was monitored with an excitation wavelength between 530–540 nm and an emission wavelength between 585–595 nm, using a multiplate reader (Synergy H1, Biotek Instruments Winooski, USA). SC-560 and DuP-697 inhibitors were used as positive controls to COX-1 and COX-2 assay, respectively.

### 2.5. 20S Proteasome Inhibition

Proteasome activity was measured by adapting a method described by Silva et al. [26]. The substrate used to study chymotrypsin-like enzyme activity was Suc-Leu-Leu-Val-Tyr-AMC and the enzyme used was purified 20S proteasome isolated from erythrocytes (Enzo Life Sciences). Briefly, Suc-Leu-Leu-Val-Tyr-AMC (25 µL of a 160 µM solution in Tris-HCl) was added to a solution containing naproxen–peptide conjugate (required amount) and 20S proteasome (70 ng) in Tris-HCl assay buffer (75 L), in a black 96-well plate. The plate was incubated at 37 °C in the dark for 2 h. The inhibition of 20S proteasome was then measured at 340 nm absorption and 460 nm emission in a microplate reader (Synergy H1, Biotek Instruments Winooski, USA). Lactacystin was used as positive control. 

### 2.6. 26S Proteasome Inhibition

This assay was carried out in the same way as described for 20S proteasome (*vide supra*), except using purified 26S proteasome isolated from AGS cells [27]. To extract 26S proteasome from cells, they were centrifuged at 1300 rpm for 3 min at 37 °C and the supernatant was rejected. The pellet was then resuspended in 5 mL of HBSS. After this step, the cells were centrifuged again under the conditions referred to above and the supernatant was rejected. Then, 1 mL of cell lysis buffer was added, before being placed in ice (4 °C) for 30 min. Subsequently, the mixture was centrifuged at 14,000 g during 30 min at 4 °C. The pellet was rejected, and the supernatant was kept in an Eppendorf tube. The quantity of protein isolated was determined by Bradford Assay.

### 2.7. Bradford Assay

A stock solution of bovine serum albumin (BSA) (1.0 mg/mL) was prepared in H_2_O. From the stock solution, six concentrations were prepared: 5%, 4%, 3%, 2%, 1% and 0% stock solution in H_2_O. An aliquot of each sample (40 µL) was added to a solution of the Bradford reagent (200 µL). After 5 min, the absorbance was measured at 595 nm and the calibration curve was plotted (correlation > 0.99). Proteasome 26S (40 mL of a sample of unknown concentration) was added to a solution of the Bradford reagent (200 mL). After 5 min, the absorbance was measured at 595 nm and the result was compared with the correlation curve in order to calculate the concentration of protein.

### 2.8. Cell Culture

Adenocarcinoma gastric cells (AGS; Sigma-Aldrich, St. Louis, MO, USA), murine-macrophage cell line (RAW 264.7; American Type Culture Collection, LGC Standards S.L.U., Barcelona, Spain), and human foetal lung fibroblasts (MRC-5; ECACC, Porton Down Salisbury, UK) were cultured as a monolayer at 37 °C in a humidified incubator with 5% carbon dioxide. AGS and RAW 264.7 cells were grown in DMEM, supplemented with 1% streptomycin/penicillin and 10% FBS (Gibco^®^). MRC-5 cells were grown in MEM, supplemented with 1% streptomycin/penicillin and 10% FBS. 

#### 2.8.1. 3-(4,5-Dimethylthiazol-2-yl)-2,5-Diphenyltetrazolium Bromide (MTT) Reduction Assay

Cell viability was evaluated by the MTT reduction assay [28]. Cells were cultured in 96-well plates (15,000 cells/well for AGS, 25,000 cells/well for RAW and 20,000 cells/well for MRC-5) and allowed to attach for 24 h. After incubation with compounds for 24 h, MTT (0.5 mg/mL final concentration) was added to each well and the plate was incubated for 75 min at 37 °C. Formazan crystals were dissolved by the addition of a DMSO: isopropanol mixture (3:1) and then quantified spectrophotometrically at 570 nm using a microplate reader (Multiskan Thermo Fisher Scientific Oy, Vantaa, Finland).

#### 2.8.2. Evaluation of NO Levels 

RAW 264.7 cells were cultured in 96-well plates (35,000 cells/well) for 24 h and then pre-treated with different concentrations of each compound. After 2 h, LPS was added (final concentration 1 µg/mL) and the plates were incubated at 37 °C, in a humidified atmosphere of 5% CO_2_. After 22 h, 75 µL of cell supernatant was transferred to a 96-well plate and mixed with 75 µL of Griess reagent (1% sulfanilamide and 0.1% naphthylethylenediamine dihydrochloride in 2% H_3_PO_4_). The plate was incubated at room temperature for 10 min in the dark and then the absorbance was read at 540 nm using a microplate reader (Multiskan Thermo Fisher Scientific Oy, Vantaa, Finland). 

### 2.9. Statistical Analysis

Statistical analysis was performed using GraphPad Prism 6 software (San Diego, CA, USA). A Shapiro–Wilk normality test was conducted to evaluate the distribution of the data, and a Grubb’s test was used to determine the presence of outliers. One-way analysis of variance (ANOVA) and Sidak’s multiple comparison test were used to determine the statistical significance between treated and untreated cells. All experiments were performed in duplicate or triplicate with at least three independent assays. Data are expressed as the mean values ± standard deviation. In all cases, values of *p* ≤ 0.05 were considered statistically significant. 

Additionally, in order to compare the results of this library of compounds and these results with other previously published, the compound dose causing 50% of enzyme/cell growth inhibition (IC_50_—half maximal inhibitory concentration) was calculated. This value allows the comparison of the effectiveness of the substances.

### 2.10. Docking Studies

The crystal structure of *Ovis aries* COX-1, expressed in *Spodoptera frugiperda* (PDB code: 3N8Z) [29], *Mus musculus* COX-2, expressed in *Spodoptera frugiperda* (PDB code: 3NT1) [30], and *Saccharomyces cerevisiae* yeast 20S open gate proteasome (PDB code: 3MG8) [31] were used as the protein receptor model. The optimized geometries of the hydrogelators ground state were obtained from ab initio molecular quantum chemistry calculations, with Gaussian 09 software and use of a 6-31+G(d,p) basis set at the DFT B3LYP level of theory. Docking of the receptor protein with the hydrogelators was performed using AutoDock4.2 suite of programs with Lamarckian Genetic Algorithm. The calculation was set up to 150 runs, 270,000 maximum number of generations, 2,500,000 maximum number of energy evaluations, and 50 50 50 grid points for proteins with 0.375 Å spacing. The macromolecule was kept rigid and ligand molecules were flexible. Visualization of the complex protein–ligand interactions was analysed with PyMOL software.

## 3. Results

To follow-up on the initial anti-inflammatory results obtained for dehydrodipeptide-naproxen conjugates **1** and **2**, a more detailed biological study of a focused library of naproxen-dehydrodipeptides (**1**–**8**) (Figure 2) was conducted, regarding their anti-inflammatory and anti-cancer activity, as well as their potential toxicity towards non-cancer cells. The synthesis of hydrogelators **1**–**8** and the rheological and physical-chemical characterization of their hydrogels was reported elsewhere [18]. Here, we report for the first time the anti-inflammatory and anti-cancer properties of conjugates **3**–**8** (Figure 2). We had previously reported the anti-inflammatory properties of compounds **1** and **2**, but no results related to their effect on cancer cells or proteasome inhibitory activity [23]. Some previous biological results for compounds **1** and **2** will be presented alongside the results for compounds **3**–**8**, where they are considered useful for comparison purposes. 

The importance of the *N*-terminal canonical amino acid residue was investigated by variation of the amino acid side chain. Studying the biological activities of **1** and **3**–**6** would allow the SAR at this position to be determined. Additionally, a comparison of compound **8** with compound **3**, where the ΔPhe residue is swapped for a ΔAla residue, would give an insight into the effect of the dehydroamino acid residue on the biological activity. A comparison of the biological results obtained for compound **7** with the results previously published for compound **1**, where the free carboxylic acid group is replaced by an ester, would give an insight into the SAR of modifications at the *C*-terminus. 

### 3.1. Anti-Inflammatory Activity

The anti-inflammatory activity of naproxen–dehydrodipeptide conjugates was investigated using a murine–macrophage cell line (RAW 264.7). Macrophages play a key role in inflammation, by initiating the immune response against invaders. Therefore, when activated by LPS, the chosen cell line can release pro-inflammatory cytokines and chemokines that lead to the production and secretion of reactive oxygen species and reactive nitrogen species, such as ^•^NO. ^•^NO levels can be measured to determine the potential of the compounds in regulating an inflammatory response. The choice of this specific macrophage cell line is based on the fact that this is one of the most used cell lines for anti-inflammatory activity studies [32,33,34], which allows us to compare our results with other available results.

#### 3.1.1. Effect of the Compounds on the Viability of RAW 264.7 Macrophages

**Figure 3:**
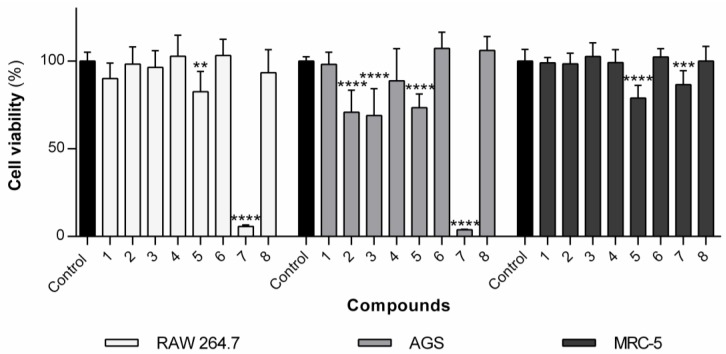
Cell viability of RAW 264.7, AGS and MRC-5 in the presence of compounds **1**–**8** at 100 µM for 24 h. Values are shown with mean ± SD. ** *p* ≤ 0.01, *** *p* ≤ 0.001, **** *p* ≤ 0.0001. The results for compounds **1** and **2** on the viability of RAW 264.7 have been reported previously but are included here for comparison purposes.

The library of naproxen–dehydrodipeptide conjugates shown in Figure 2 was tested for their effect on the viability of rat macrophages (RAW 264.7 cells) (Figure 3). Compound **5** elicited a small cytotoxic effect at 100 µM. Compounds **3**, **4**, **6** and **8** did not show any statistically significant cytotoxic effects on RAW cells up to 100 µM, as was the case with the previously studied compounds **1** and **2** [23]. Compound **7**, containing a methyl ester at the *C*-terminus, was found to be cytotoxic to some degree, with an IC_50_ value of 23.1 µM, suggesting that it could cross the cell membrane and elicit cellular toxicity, presumably following ester hydrolysis by intracellular esterases [35].

#### 3.1.2. Effect of the Compounds on the Production of ^•^NO in RAW 264.7 Macrophages

**Figure 4:**
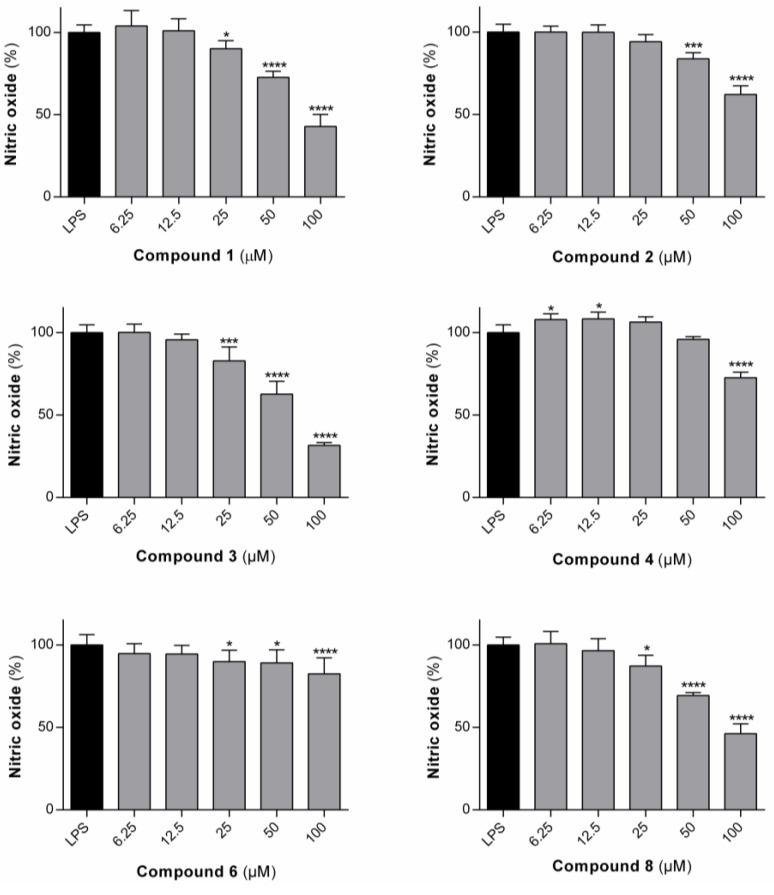
LPS-induced ^•^NO production in rat macrophages in the presence of the compounds **1**–**4**, **6** and 8 for 24 h. Values are shown with mean ± SD. * *p* ≤ 0.05; *** *p* ≤ 0.001; **** *p* ≤ 0.0001. The results for compound **1** and **2**, reported previously, are included for comparison purposes.

The compounds which were shown to be non-toxic to rat macrophages (**3**, **4**, **6** and **8**) were tested for their ability to inhibit LPS-dependent ^•^NO production in rat macrophages (Figure 4). ^•^NO is an important mediator of the inflammatory response, which is synthesized by inducible nitric oxide synthase (iNOS) from oxygen and L-arginine [36]. Its excessive production is associated with inflammatory diseases [37]. The dehydrodipeptides generally elicited only modest effects on the production of ^•^NO. IC_50_ values of 64.7 µM and 84.4 µM were determined for the most active compounds, **3** and **8**, respectively, in line with the IC_50_ value of 79.3 µM, previously reported for compound **1** [20]. 

#### 3.1.3. Effect of the Compounds on LOX Activity

**Figure 5:**
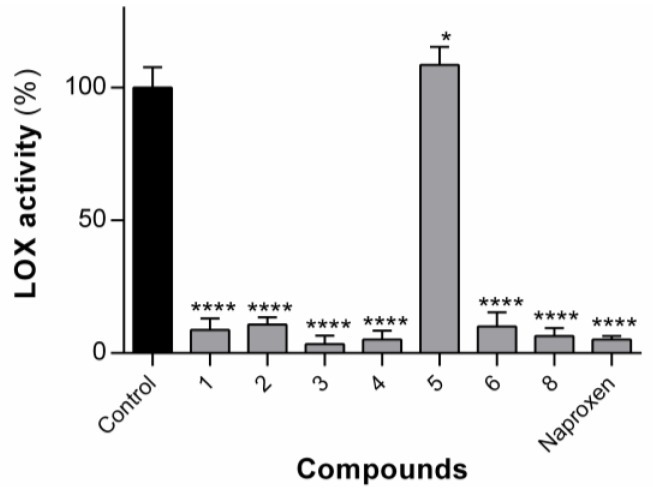
LOX activity in the presence of compounds **1**–**6** and **8** at 100 µM. Values are shown with mean ± SD. **** *p* ≤ 0.0001. The results for compound **1** and **2**, reported previously, are included for comparison purposes.

The compounds **1**–**6** and compound **8** were tested for their ability to inhibit the arachidonic-pathway-related 5-LOX enzyme (Figure 5). Compound **7** could not be tested, owing to insolubility in the assay buffer solution. The LOX enzyme is responsible for the production of inflammatory leukotrienes, which are a major cause of inflammation in asthma, allergic rhinitis and osteoarthritis [38]. The compounds were tested at single concentrations of 100 µM in the first instance. As reported previously for dehydrodipeptides **1** and **2** [23], compounds **3**, **4**, **6** and **8** at 100 µM concentration were also able to significantly inhibit the LOX enzyme to a similar level to naproxen. Compound **5** was not able to inhibit LOX activity. 

Aside from compound **5**, the activity seems relatively insensitive to modifications at both the *N*-terminal canonical amino acid residue and the *C*-terminal dehydroamino acid residue. Compound **5**, containing an *N*-terminal charged lysine residue, is the most polar compound of the set (cLogP = 3.89) and therefore might not be able to mimic the hydrophobic interactions of the fatty acid chain of the natural substrate (arachidonic acid) with the LOX enzyme active site. The other compounds in this series contain either non-polar (Ala for **4**, Met for **6**) or amphipathic (Tyr for **1**, Trp for **3**) amino acid residues.

The most active compounds, **3**, **4** and **8**, as well as **1** and **2**, were subsequently tested in a dose–response assay in order to calculate their IC_50_ values (Table 1). All compounds afforded IC_50_ values significantly higher than was observed for the parent molecule, naproxen, presumably reflecting a limited ability of the LOX binding site to accommodate the larger structure of the conjugates. Compounds **1**, **3**, **4** and **6** display similar IC_50_ values, highlighting again that the *N*-terminal amino acid side chain can be modified without a deleterious effect on activity. 

#### 3.1.4. Effect of the Compounds on COX-1/COX-2 Activity

**Figure 6:**
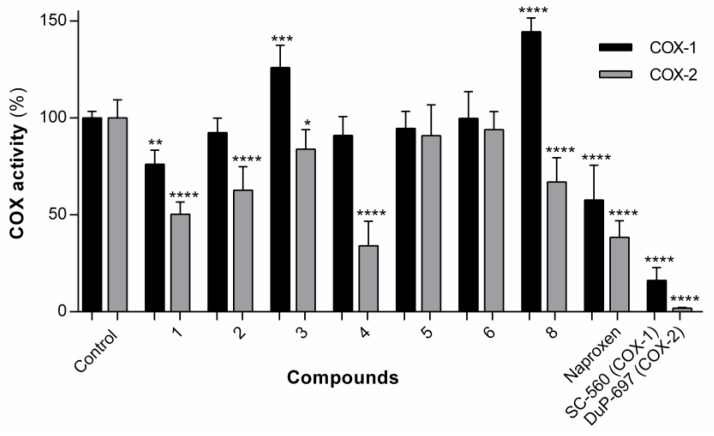
COX-1 and COX-2 activities in the presence of compounds **1**–**6** and **8** at 25 µM. Values are shown with mean ± SD. * *p* ≤ 0.05; ** *p* ≤ 0.01; *** *p* ≤ 0.001, **** *p* ≤ 0.0001. The results for compounds **1** and **2** have been reported previously but are included here for comparison purposes.

The library of naproxen–dehydrodipeptide conjugates was tested for their ability to inhibit the COX-1 and COX-2 isozymes (also known as prostaglandin–endoperoxide synthase) (Figure 6). Compound **7** was insoluble in the assay medium. 

COX is an enzyme that catalyses the formation of pro-inflammatory prostanoids, such as thromboxane and prostaglandins, from arachidonic acid. The inhibition of COX enzymes can provide relief from inflammation and pain. The drugs aspirin and ibuprofen work by unselectively inhibiting both COX-1 and COX-2 isozymes. Recently, the search has switched towards selective COX-2 inhibitors which can exert their effects without the gastrointestinal side-effects that can be caused by COX-1 inhibition. The structures of the two isozymes are very similar, but nonetheless many selective COX-2 inhibitors have been identified, such as celecoxib, rofecoxib and etoricoxib [39]. In this assay, we measured COX inhibition using a commercially available kit, which utilizes the peroxidase component of COX enzymes and is based on the reaction between prostaglandin G2 (PGG2) (product of COX activity) and ADHP, which produces a highly fluorescent compound, resorufin.

In general, the compounds displayed higher levels of COX-2 inhibition than COX-1 inhibition, although overall it would seem that conjugation with dehydrodipeptides interferes with the inhibitory capacity of naproxen to some degree. The exception, and the most interesting compound, is compound **4**, which displays significant COX-2 inhibition (66.0% inhibition at 25 µM), similar to the parent compound, naproxen (61.5% inhibition at 25 µM), but with low levels of COX-1 inhibition (9.0% at 25 µM) compared with the parent compound, naproxen (42.3% at 25 µM). Therefore, the structure of compound **4** is a potential lead towards a selective COX-2 inhibitor. 

**Figure 7:**
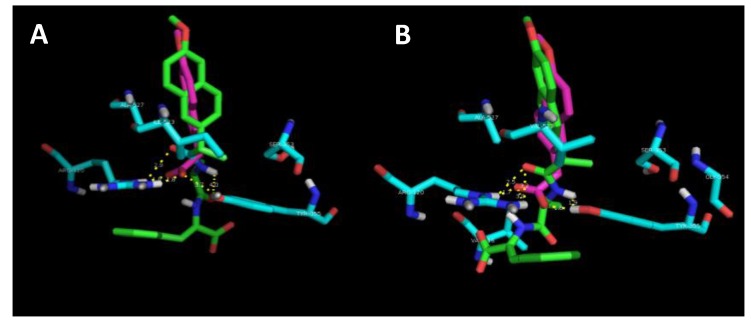
Images for the best docking conformations (lowest interaction energy) of compound **4** (green) and naproxen (pink) in the binding site of COX-1 (**A**) and COX-2 (**B**) isozymes. The amino acid residues which interact with the inhibitors are coloured blue and the hydrogen bonds are represented as yellow dashed lines.

A preliminary docking study was performed to provide an insight into the interaction of the naproxen–dehydrodipeptide scaffold with the active site of the COX-1 and COX-2 isoenzymes [29,30] (Figure 7, Table 2). The crystal structures of COX-1 and COX-2 enzymes show a high degree of sequence homology (~60%), but, nevertheless, there are key differences between the active sites [39,40]. The Ile-434 and Ile-523 residues of COX-1 are switched for smaller valine residues in COX-2 [39]. In addition, the His-513 residue of the COX-1 isozyme is switched for an arginine residue [39]. 

In the lowest energy binding conformations, naproxen binds to the active site main channel of COX-1 and COX-2 isoenzymes in a similar manner, with the carboxylic acid group establishing hydrogen bonds with the Arg120 and Tyr355 residues, as seen for the binding of arachidonic acid. The naphthalene moiety of naproxen is also stabilised by hydrophobic and *π*–*π* stacking interactions with apolar residues lining the active site of the main channel. The slightly more favourable binding energy calculated for the interaction of naproxen with COX-2 is in line with the extra space created in the active site main channel by the replacement of the Ile523 residue in COX-1 by the Val523 residue in COX-2. The naproxen moiety of compound **4** binds in the active site main channel of the COX isozymes in a similar fashion to naproxen. The amide bond connecting the naproxen moiety to the *N*-terminal amino acid in compound **4** establishes hydrogen bonds with the Arg120 and Tyr355 residues in a similar way as seen for the carboxylic acid group of naproxen. The narrower main channel of COX-1 seems to induce a rotation of the naproxen moiety of compound **4**, that presumably results in weaker hydrophobic interactions with the main channel residues and also in longer (weaker) hydrogen bonds between the amide group of compound **4** and the Arg120 and Tyr355 residues of COX-1. Additionally, the C-terminal dehydrophenylalanine residue of compound **4** seems to occupy a binding pocket lined by His and Pro residues in COX-2, while in COX-1 this residue seems to be mainly exposed to the solvent. The combination of different ensembles of molecular interactions results in a significant selectivity of compound **4** towards COX-2, as measured experimentally and reproduced by the docking studies. 

The experimental results suggest show that the nature of the canonical amino acid side chain effects the level of COX inhibition. More specifically, compound **4**, containing an alanine residue and thus the smallest amino acid side chain (methyl) is able to significantly inhibit COX-2 without inhibiting COX-1. From the modelling study, it would seem that optimal binding of the naproxen–dipeptide conjugate would occur when the *N*-terminal amino acid side chain can fit into a narrow binding pocket formed by the Ser353 residue present in both COX isozymes. It follows that most of our compounds contain an amino acid side chain that is too sterically demanding to be easily accommodated by this narrow binding pocket, and therefore most compounds elicit only weak inhibition of COX-1 and COX-2 isozymes. Compound **4** is the exception, and possesses only a small methyl amino acid side chain. This methyl side chain may be close a threshold steric size, able to be accommodated by the binding pocket of the COX-2 isozyme, but not still not quite be accommodated by the binding pocket of the COX-1 isozyme, resulting in a selective inhibition of COX-2. It would be interesting to see if switching the alanine residue for a glycine residue would further enhance or erode COX-2 selectivity.

### 3.2. Anti-Cancer Activity

#### 3.2.1. Effect of the Compounds on Cell Viability of AGS Cells

**Figure 8:**
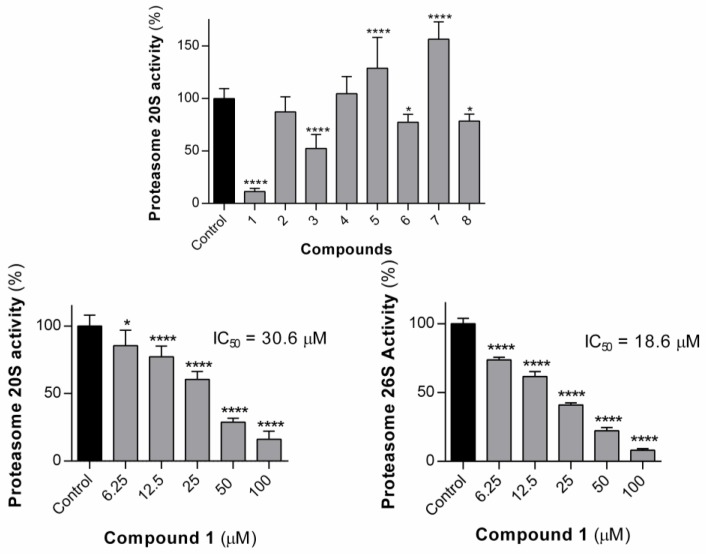
Proteasome 20S activity in the presence of compounds **1**–**8** at 100 µM and dose-response graphs for compound **1** 20S and 26S proteasome. Values are shown with mean ± SD. * *p* ≤ 0.05; **** *p* ≤ 0.0001.

The toxicity of the compounds towards the cancer cell-line AGS, was probed by performing MTT viability assays (Figure 8). The compounds **1**, **4**, **6** and **8** were not toxic to AGS cells at 100 µM, whilst **2**, **3** and **5** were found to be slightly cytotoxic at the same concentration. The only compound which showed a high toxicity was **7**, which had an IC_50_ of 10.9 µM. It would appear that the presence of the ester group is important for cytotoxicity, as the structurally related compound **1** shows no toxic effect at 100 µM, even though the structures only differ in the switching of the ester group for a carboxylic acid group. This result reinforces the suggestion that the cellular toxicity of compound **7** might result from cell membrane crossing ability, due to its uncharged nature and lipophilic nature, presumably followed by activation by cytosolic esterases.

#### 3.2.2. Effect of the Compounds on Proteasome Activity

**Figure 9:**
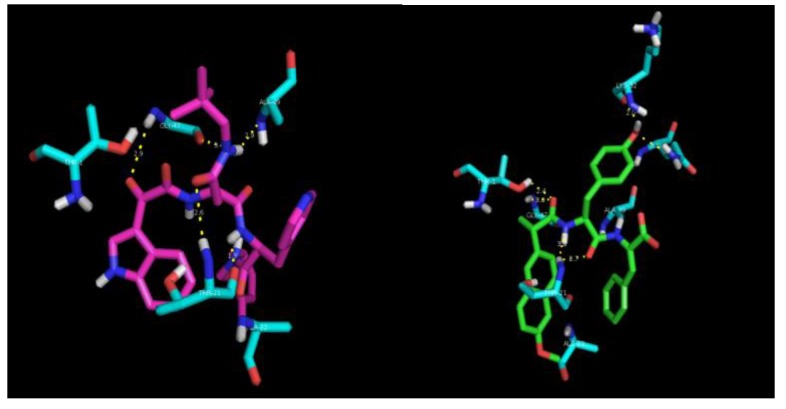
Best docking poses for an optimised non-covalent inhibitor (pink) of proteasome 20S and for compound **1** (green). The protein residues which interact with the inhibitors are coloured blue and the hydrogen bonds are represented by yellow dashed lines.

As mentioned previously, the library of naproxen–dehydrodipeptide conjugates can be studied for the modulating effect of the peptide on the cognate naproxen pharmacological properties or studied as novel chemical entities with novel properties. As proof-of-concept, the compound library was tested for their ability to inhibit proteasome activity (Figure 9). Proteasomes play an important regulatory role, catalysing the degradation of misfolded proteins [41]. Misfolded proteins are first polyubiquitinated, and then proceed through a complex cascade of reactions before being hydrolysed by the proteasome. The proteasome system is of interest for cancer therapy because cancer cells have a faster rate of metabolism that is more sensitive to problems with proteasome function, and will die more quickly if the degradation system is interrupted [42]. The 26S proteasome, located in the cytoplasm and the nucleus of eukaryotic cells, consists of three subunits, two regulatory (19S) subunits and one catalytic (20S) subunit [42]. The 19S subunits are located at either end of the barrel-shaped 20S subunit, and carry out the process of deubiquitination of a target protein, which will then pass to the 20S subunit to be degraded [42]. We considered compounds **1**–**8** as potential proteasome inhibitors for two reasons: (1) dehydropeptides have been shown to be useful structural units of peptidomimetic inhibitors, owing to their ability to reduce the conformational flexibility and also to increase the proteolytic stability of a peptide chain [12,43] and (2) the molecules bear a structural resemblance to the known proteasome inhibitor and multiple myeloma drug, bortezomib (and related analogues) [44]. 

We tested our compounds against the 20S subunit in the first instance, at single concentrations of 100 µM. Most of the compounds proved to be inactive or almost inactive, the exceptions being compounds **1** and **3**, which reduced enzyme activity to 11% and 52%, respectively, at this concentration. Structurally, the two active compounds, **1** and **3**, share a common feature, in that they are the only two compounds to feature aromatic groups on both the dehydroamino acid residue and the central amino acid residue, suggesting binding to the chymotrypsin-like hydrolytic site of the proteasome located on the *β*-5 subunit. The most active compound **1**, was selected for a dose–response assay and was found to display an IC_50_ value of 30.6 µM. The methyl ester analogue of **1**, compound **7**, was not able to inhibit the 20S proteasome. Compound **1** was also tested for its ability to inhibit the proteasome 26S subunit. At 100 µM, dehydrodipeptide **1** reduced the 26S proteasome activity to 8.1% versus control. A dose–response assay was conducted for compound **1** in order to calculate the IC_50_ value, which was shown to be 18.6 µM. It is interesting that compound **1** is able to inhibit the 26S proteasome more than it does the 20S proteasome. The 26S proteasome is a 20S subunit with one or two 19S capping units attached, suggesting that **1** might be binding to the 19S subunit. There is precedent in the literature for peptides containing dehydroamino acid residues being able to inhibit the 19S subunit. Thiostrepton, for example, is able to inhibit yeast proteasome through covalent modification of a cysteine residue of the 19S subunit [20].

As dehydrodipeptides are putative Michael acceptors, we decided to test the hypothesis that proteasome inhibition by compound **1** could result from covalent modification of the nucleophilic residue of the chymotripsyn-like hydrolytic site. The crystal structure of bortezomib, bound to yeast 20S proteasome [45], shows that the terminal boronic acid group of bortezomib forms a reversible covalent bond with Thr1 within the active site. 

A docking study was conducted in order to obtain an insight into the molecular interactions between the chymotrypsin-like hydrolytic site of yeast proteasome 20S and dehydrodipeptide **1** (Figure 9).

As can be seen, the peptide scaffold of compound **1** (green) and of an optimized noncovalent inhibitor (pink) of the proteasome [31] display similar hydrogen bonding interactions with the proteasome catalytic site amino acid residues Thr1, Th21, Gly47 and Ala49. In the best docking conformation of compound **1**, the dehydrophenylalanine residue is far too remote from the nucleophilic Thr1 chain to permit a chemical reaction. Thus, one must assume that compound **1** is a noncovalent reversible inhibitor of the proteasome.

### 3.3. Effect of the Compounds on the Cell Viability of MRC-5 Cell Line

In order to assess the potential toxicity of the molecules under study towards non-cancer human cells, the human lung fibroblast cell line MRC-5 was used (Figure 3). In general, the molecules presented little to no toxicity. No significant decrease in cell viability was observed for compounds **1**–**4**, **6** and **8** at the highest concentration tested (100 µM). Compounds **5** and **7** exhibited some cytotoxicity to MRC-5 cells at the highest concentration tested (100 µM), with observed viabilities of ca. 79% and 87%, respectively. Compound **5** has a similar structure to the non-toxic analogous conjugates **1**, **3**, **4** and **6**. The toxicity of compound **5** may be ascribed to the protonated amine side chain group on the *N*-terminal Lys residue, which is known to promote membrane permeability. 

## 4. Discussion

In the present study, we have investigated the ability of naproxen–dehydrodipetide hydrogelators to act as potential therapeutic agents in their own right, with the added appeal of direct topical application, which might allow for lower doses and fewer side-effects. To this end, the panel of conjugates **1**–**8**, where the amino acid side chains had been varied, were evaluated for their biological activity and cytotoxicity. Their potential anti-inflammatory and anti-cancer activities, as well as their effect on non-cancer cells, were assessed using a variety of enzymatic and cellular assays. In general, the SAR of anti-inflammation was relatively insensitive to the structure of the central amino acid, perhaps suggesting that the anti-inflammatory activity observed is principally derived from the naproxen residue, and that the appended peptide serves to tune the biological behavior. Therefore, in most cases, the compounds might be considered as naproxen conjugates where a more biologically inert dipeptide serves to confer hydrogelator properties onto the active part of the molecule, potentially allowing topical application. However, there were a few cases where the dehydrodipeptide unit was able to favorably modify the biological activity. For example, in the COX enzymatic assay, compound **4** was able to inhibit the COX-2 isozyme at a greater level than naproxen, without inhibiting COX-1, and therefore is a potential lead in the search for selective COX-2 inhibitors (naproxen itself is a mixed COX-1/COX-2 inhibitor). In LOX assay, all analogues except **5** were all able to significantly inhibit the LOX enzyme at a similar level to naproxen. Compound **4**, displaying both high selectivity for COX-2 inhibition and high levels of LOX inhibition, is a potential candidate for dual COX/LOX inhibition as an optimised strategy for treating inflammatory conditions. Compound **1** was the only analogue to significantly inhibit proteasome 20S activity, and this compound was also able to inhibit the 26S proteasome, despite naproxen itself being reported to not be an inhibitor of proteasome [27,41], suggesting that the observed activity arises from the peptide portion of the molecule. Its methyl ester analogue, **7**, was inactive against proteasome. Compound **7** was, however, the only compound to be active against a gastric adenocarcinoma cell line (AGS) at low concentrations. Most of the compounds were non-toxic to human cells (MRC-5) and macrophage cells (RAW 264.7). Overall, these compounds are promising leads for the development of anti-inflammatory hydrogelators for topical application. 

## 5. Conclusions

A panel of dihydroamino acid–amino acid–naproxen conjugates have been evaluated for their biological activity. In general, the compounds are able to strongly inhibit the activity of the LOX enzyme, at a similar level to naproxen, with the level of inhibition being insensitive to the nature of the central amino acid, provided the side chain does not contain a charged group. In a COX enzymatic assay, compound **4** (possessing the smallest amino acid side chain of the panel, Me) was the most interesting, showing a high level of inhibition of the COX-2 enzyme (more active than naproxen itself) whilst being inactive against the COX-1 enzyme, and is therefore a promising lead in the search for selective COX-2 inhibitors. When these results are taken together, compound **4** is a potential candidate for dual COX/LOX inhibition in optimised NSAID anti-inflammatory treatment. Compound **1** was the most active compound in the proteasome assays. Generally, most of the compounds show a low toxicity to MRC-5, except for compound **7**, which was also the most active against the cancer cell line AGS. Taken as a whole, this class of compound warrants further study, particularly as anti-inflammatory hydrogels where topical application might allow targeted therapy.

## Figures and Tables

**Table 1:** IC_50_ values for LOX inhibition.

Compound	IC_50_ (µM)
1	54.1
2	67.4
3	55.9
4	55.7
6	60.3
8	48.9
Naproxen	22.0

**Table 2:** Binding energy for the best docking conformations of compound **4** and naproxen in the binding site of COX-1 and COX-2 isozymes.

Binding Energy (kcal/mol)	COX-1	COX-2
Compound **4**	−7.52	−8.4
Naproxen	−8.22	−8.95

## Abbreviations

NSAIDs	non-steroidal anti-inflammatory drugs
SPIONs	superparamagnetic iron oxide nanoparticles
MRI	magnetic resonance imaging
COX-2	cyclooxygenase-2
COX-1	cyclooxygenase-1
COX	cyclooxygenase
LPS	lipopolysaccharide
LOX	lipoxygenase
^•^NO	nitric oxide
SAR	structure-activity relationship
DMSO	dimethyl sulfoxide
MTT	*N*-(1-naphthyl)ethylenediamine, 3-(4,5-dimethylthiazol-2-yl)-2,5-diphenyltetrazolium bromide
DMEM	Dulbecco’s Modified Eagle Medium
MEM	Minimum Essential Medium
FBS	foetal bovine serum
BSA	bovine serum albumine
PGG2	prostaglandin G2
ADHP	10-acetyl-3,7-dihydroxyphenoxazine
